# The Connectivity Fingerprints of Highly-Skilled and Disordered Reading Persist Across Cognitive Domains

**DOI:** 10.3389/fncom.2021.590093

**Published:** 2021-02-12

**Authors:** Chris McNorgan

**Affiliations:** Department of Psychology, University at Buffalo, Buffalo, NY, United States

**Keywords:** functional connectivity, dyslexia, cognition, machine learning, math cognition, learning disabilities, fMRI

## Abstract

The capacity to produce and understand written language is a uniquely human skill that exists on a continuum, and foundational to other facets of human cognition. Multivariate classifiers based on support vector machines (SVM) have provided much insight into the networks underlying reading skill beyond what traditional univariate methods can tell us. Shallow models like SVM require large amounts of data, and this problem is compounded when functional connections, which increase exponentially with network size, are predictors of interest. Data reduction using independent component analyses (ICA) mitigates this problem, but conventionally assumes linear relationships. Multilayer feedforward networks, in contrast, readily find optimal low-dimensional encodings of complex patterns that include complex nonlinear or conditional relationships. Samples of poor and highly-skilled young readers were selected from two open access data sets using rhyming and mental multiplication tasks, respectively. Functional connectivity was computed for the rhyming task within a functionally-defined reading network and used to train multilayer feedforward classifier models to simultaneously associate functional connectivity patterns with lexicality (word vs. pseudoword) and reading skill (poor vs. highly-skilled). Classifiers identified validation set lexicality with significantly better than chance accuracy, and reading skill with near-ceiling accuracy. Critically, a series of replications used pre-trained rhyming-task models to classify reading skill from mental multiplication task participants' connectivity with near-ceiling accuracy. The novel deep learning approach presented here provides the clearest demonstration to date that reading-skill dependent functional connectivity within the reading network influences brain processing dynamics across cognitive domains.

## The Connectivity Fingerprints of Highly-Skilled and Disordered Reading Persist Across Cognitive Domains

Proficient reading is a prerequisite for formal instruction and independently navigating the world, but is a skill that exists on a continuum. Developmental dyslexia is a learning disorder characterized by a difficulty in reading in the absence of any other pronounced cognitive difficulty, and is the most commonly diagnosed learning disorder (Shaywitz and Shaywitz, 2005). By definition, children with specific reading disability possess normal-range intelligence, though reading difficulty is often comorbid with dyscalculia. The factors underlying learning difficulty in both domains is not well understood, but it has been proposed that it may be attributable to a shared reliance on core cognitive processes (Archibald et al., 2013). At the other end of the continuum of reading skill, precocious reading is associated with a modest advantage in other language abilities in later childhood (Mills and Jackson, 1990), but it has not been shown to confer a general cognitive advantage in other domains.

Reading maps visual to phonological representations, and is thus fundamentally an audiovisual process. An extensive literature has explored neural processing in dyslexics and typically developing readers, and points to a model in which reading difficulty is attributable to disordered audiovisual integration of orthographic and phonological representations (Richlan, 2019). Relative to controls, dyslexics under-activate the left temporo-parietal cortex (Temple et al., 2001), and show delayed specialization in the ventral visual object processing stream for visual word processing (van der Mark et al., 2009). Under-activation in this network of regions has been argued to reflect failure of audiovisual integration processing (Blau et al., 2010; Randazzo et al., 2019) and failure to engage lexical-semantic representations (Richlan et al., 2009). Relative to controls, dyslexics over-activate the right occipitotemporal cortex and anterior inferior temporal gyrus, which has been suggested to reflect compensatory activation (Shaywitz and Shaywitz, 2005). This set of anatomically distributed brain regions supporting orthographic, phonological and semantic processing of written language is referred to as the reading network (Perfetti et al., 2007; Perfetti and Tan, 2013; Morken et al., 2017). All contemporary brain-based models of fluent and disordered reading assume that reading entails interactions within this network of more-or-less functionally-specialized brain regions (e.g., Dehaene et al., 2010; Price and Devlin, 2011).

If patterns of regional activation within this network are the dynamic product of connectivity among these regions, connectivity differences between skilled and poor readers must underlie the group differences described above. Dyslexic readers show weaker reading task-based functional connectivity between the visual word form area and other regions within the left hemisphere reading network, but greater connectivity between the visual word form area and left middle temporal and middle occipital gyrus (van der Mark et al., 2011). Left angular gyrus has also been implicated as a critical hub, with reduced task-based functional connectivity with other reading network nodes for dyslexics, but increased connectivity to posterior right hemisphere, possibly attributable to compensatory recruitment during phonological tasks (Pugh et al., 2000).

### Multivariate Studies of Normal and Disordered Reading

All brain-based reading models agree that fluent reading entails cooperation among regions within the reading network that may be only conditionally involved (e.g., when the task involves phonological assembly, as in Pugh et al., 2000). Nonetheless, models are largely informed by a literature that relies on univariate general linear model analyses (GLMA), which are limited in two important respects: First, they assume linear relationships between observed and modeled values, requiring multiple independent hypothesis tests to identify conditionally-involved regions or connections. Second, because univariate analyses examine only local relationships, they cannot incorporate informative contextual information from other sources. To address the second concern, multivariate analyses have been increasingly important for informing the literature.

Multivariate pattern analyses (MVPA) commonly use machine learning classifiers to decode patterns of activity among voxel populations, revealing regional categorical sensitivity that may not manifest as category-dependent mean differences in response amplitude in a conventional GLMA. Models of normal and disordered reading have been refined by MVPA in studies, showing, for example, that impaired access to phonological representations, rather than distorted representations, may underlie reading difficulty (Boets et al., 2013; Norton et al., 2015). MVPA have also shown that regional gray matter volume patterns in posterior occipito-temporal and temporal-parietal cortices differ between dyslexics, typical readers and those with specific reading comprehension deficit (Bailey et al., 2016). The enhanced sensitivity of MVPA was leveraged in a whole-brain fMRI analysis of longitudinal data using recursive feature elimination to find that dyslexics who made gains in reading skill over a 2.5 year period could be discriminated from those who did not on the basis of activity among voxels in right IFG, left prefrontal cortex and left temporoparietal cortex (Hoeft et al., 2011).

Multivariate approaches have also been applied at the network-level. Wolf et al. (2010) used a multivariate independent components analysis (ICA) to examine network-level functional connectivity differences in older adolescents using a working memory task, arguing ICA-based methods are better-able to detect distributed network components (Esposito et al., 2006). Their analysis found that the working memory delay period was associated with increased connectivity for dyslexics within a left-lateralized frontoparietal network, and mixed differences in a second bilateral frontoparietal network, both including regions implicated in phonological processing, which they argue may reflect increased reliance on working memory in dyslexics during phonological processing. A later ICA network analysis found that improvements in word reading and comprehension following reading remediation were correlated with connectivity changes in functional networks supporting visual attention and executive control (Horowitz-Kraus et al., 2015). Multivariate studies have thus refined a model of skilled and disordered reading as dependent on interactions among multiple cognitive systems, and not reliant on a single system.

The univariate and multivariate approaches described above rely on the general linear model to test relationships between observed and predicted values for one or more variables. However, linear models may be good approximations only within certain ranges, as all biological systems exhibit nonlinearity (e.g., saturation) along some range of inputs (Korenberg and Hunter, 1990). Conditional relationships are an important class of nonlinear relationships, and though they can often be linearly modeled—for example by partitioning a continuum into groups and demonstrating a nonremovable interaction—doing so requires an experiment designed around testing the interaction.

### Multilayer Feedforward Classifiers

Support vector machines are the most commonly applied machine learning application in MVPA (Mokhtari and Hossein-Zadeh, 2013). These kernel-based approaches can use nonlinear kernels to form classification boundaries, but require advanced knowledge of a suitable nonlinear kernel function. The multilayer feedforward classifier is an alternative machine learning approach that uses the backpropagation of error algorithm (Rumelhart et al., 1985) to discover an optimal nonlinear classification function. Though these models are arguably more difficult to interpret than are their simpler counterparts (Norman et al., 2006), they have several advantages: First, they are extremely powerful, and with the development of training algorithms and architectures that mitigate concerns associated with high-dimensional data (Poggio et al., 2017), variations of these networks have been foundational to the recent *Deep Learning* renaissance in machine learning. Second, they are arbitrarily flexible, with the capacity to accommodate multiple outputs. This feature allows these networks to find a solution space that best fits the training data with respect to multiple problem domains. McNorgan et al. (2020b) applied such a network to the classification of imagined objects from fMRI data, simultaneously learning to distinguish among object categories and identifying the functional networks supporting category processing. Because the model use shared parameters to solve each problem, the solution in one problem domain (e.g., object categorization) is explicitly tied to the other problem domain (e.g., functional connectivity), making alternative explanations more unlikely.

### The Present Study

Because there are *n*^2^ connections among *n* regions, connectivity studies of large networks face practical computational, interpretation and statistical challenges, and seed-based approaches are thus often used to restrict analyses to the *n-*1 connections to a seed region. Larger networks are not well suited for exploration using conventional parametric methods because univariate methods must avoid inflating the Type I error rate, and even multivariate methods like the MANOVA mathematically require a sample size that exceeds the number of variables. The present study uses a multilayer feedforward classifier network to explore large-network connectivity, and as a nonparametric multivariate analysis, suffers neither of these drawbacks. The analysis takes advantage of the extensibility of feedforward neural networks to simultaneously identify task-related functional connections that distinguish between poor- and highly-skilled readers and between word and pseudoword processing.

## Materials and Methods

### Archival Data

Neuroimaging and phenotypic data were obtained from two open access longitudinal datasets hosted on the OpenNeuro.org data repository, and described in detail in Lytle et al. (2019) and Suárez-Pellicioni et al. (2019). The first (“Reading Set”) comes from Lytle et al. (2019), and was collected from 188 children between the ages of 8 and 13 years spanning a range of reading ability. The Reading Set data were collected while participants engaged in rhyming judgments of pairs of sequentially-presented lexical items (monosyllabic words or pseudowords). Within each run of the task were 6 trial types: Four types of lexical items (rhyming vs. non-rhyming items that had either similar or dissimilar spelling), a fixation cross response baseline, and a nonlinguistic symbol matching judgment. This experiment was blocked by run, using multiple presentation modality (auditory, visual, audiovisual) and lexicality (words, pseudowords) conditions, and the present study analyzed only data from the unimodal visual condition.

The second (“Math Set”) comes from Suárez-Pellicioni et al. (2019), and was collected from 132 children between the ages of 8 and 14 years spanning a range of math and reading ability. These data were selected for two reasons: First, the Math Set study drew from the same population as the Reading Set study, and was carried out concurrently with the Reading Set study by the same research staff, using the same equipment and the same standardized testing procedures. Second, because mental arithmetic arguably bears little similarity to rhyming judgments of written words, validation of the classifier models against these data provides an extremely challenging test of generalizability. The Math Set experiment was blocked by run, during which participants made relative numerosity judgments, performed mental subtraction or multiplication. The present study analyzed only data from the single-digit mental multiplication runs, which was selected because single-digit multiplication is commonly taught by rote memorization and was assumed to be the mental arithmetic task most likely to involve lexical processing. Aside from domain-specific difficulties in reading or math fluency, participants in both studies were cognitively normal.

The Reading Set and Math Set studies were carried out in accordance with recommendations of the Institutional Review Board at Northwestern University and the anonymized data were released for reanalysis. The protocols were approved by the Institutional Review Board of Northwestern University. Parents of all participants gave written informed consent in accordance with the Declaration of Helsinki.

### Participant Selection

Participants were selected from among those who had completed both longitudinal time points in either dataset. This initial selection criterion was motivated by two important considerations: First, participants with poor-quality data resulting from, e.g., excessive movement, failure to satisfactorily perform the task etc., were not invited to return for the second longitudinal time point, and were thus heuristically excluded from the study. Second, the longitudinal dataset permits analytic flexibility to accommodate follow-up investigations of developmental changes. From among those participants, subsets of participants were selected from each of the datasets on the basis of MRI data quality and standardized test scores. Fourteen poor readers with low scores (< 85 scaled score) across multiple standardized measures of reading skill at the first longitudinal time point were selected from among the Reading Set participants. These participants were matched against 14 highly-skilled readers with high scores across the same standardized measures of reading skill. Aside from the poor scores in measures specific to reading ability, the poor readers had otherwise normal language ability, as indicated by their spoken word recognition and picture vocabulary scores, which were within the normal range, as were their WASI subtest and full-scale IQ scores. This selection method followed the approach applied to this dataset in McNorgan et al. (2013) which identified matched samples of 13 typically-developing readers and 13 children reaching a clinical criterion for reading-specific impairment using similar selection criteria. Standardized testing scores for the Reading Set participants are presented in Table 1. Five Math Set participants with the lowest Woodcock-Johnson III reading subtest scores (≤85) at the first longitudinal time point were matched against five participants with high scores at the first longitudinal time point on the same tests. Standardized testing scores for these participants are presented in Table 2.

**Table 1:** Mean (SD) standardized measure scores for Reading Set participants.

**Measure**	**Poor**	**Skilled**	***t*(26)**	
WJ-III WordID	89 (6)	119 (8)	11.04	
WJ-III Word Attack	91 (11)	117 (8)	9.84	
WJ-III Passage Comprehension	90 (11)	110 (12)	5.01	
WJ-III Oral Comprehension	106 (12)	112 (11)	1.29	*n.s*.
WJ-III Picture Vocabulary	112 (12)	110 (13)	−0.3	*n.s*.
TOWRE SW	92 (13)	115 (10)	5.02	
TOWRE PDE	90 (17)	118 (12)	4.76	
WASI Verbal IQ	110 (16)	123 (15)	1.77	
WASI Performance IQ	107 (12)	120 (11)	2.64	
WASI FSIQ	110 (14)	124 (11)	2.59	

*WJ-III, Woodcock-Johnson III; TOWRE, Test of Word Reading Efficiency; WASI, Wechsler Abbreviated Scale of Intelligence. All t statistics are significant at p < 0.05 unless otherwise noted (n.s.)*.

**Table 2:** Mean (SD) standardized measure scores for Math Set participants.

**Measure**	**Poor**	**Skilled**	***t*(8)**
WJ-III WordID	92 (8)	125 (5)	4.53
WJ-III Word Attack	96 (8)	114 (11)	2.07
WJ-III Passage Comprehension	91 (6)	116 (4)	4.61
WJ-III Passage Comprehension	N/A	N/A	
WJ-III Oral Comprehension	N/A	N/A	
TOWRE SW	90 (6)	124 (7)	7.58
TOWRE PDE	86 (8)	113 (18)	2.65
WASI Verbal IQ	83 (1)	143 (3)	37.4
WASI Performance IQ	89 (5)	119 (14)	4.05
WASI FSIQ	84 (3)	135 (6)	14.39

*WJ-III, Woodcock-Johnson III; TOWRE, Test of Word Reading Efficiency; WASI, Wechsler Abbreviated Scale of Intelligence. All t statistics are significant at p < 0.05*.

### Neuroimaging Data Processing

MRI acquisition details can be found in the dataset descriptors provided in Lytle et al. (2019) and Suárez-Pellicioni et al. (2019). FreeSurfer/FS-FAST (version 6.0, http://surfer.nmr.mgh.harvard.edu) was used for all fMRI data preprocessing and GLM analyses. Reading Set and Math Set data were collected from the same MRI scanner with the same acquisition parameters, and identical processing pipelines were applied to both data sets to obtain functional connectivity estimates and generate machine learning classifier patterns.

#### Anatomical Data Processing

After segmenting into white and gray matter volumes, the mean of the timepoint 1 and timepoint 2 T1-weighted images were rendered in 3D surface space and normalized to the FreeSurfer standard template space. Cortical surface space was labeled using an automated atlas-based parcellation of the gyri and sulci (Destrieux et al., 2010).

#### Functional Data Processing

We applied here the data processing pipeline used in a recent application of a multilayer machine learning classifier to functional connectivity and coarse-scale cortical pattern analysis (McNorgan et al., 2020a). Functional images for both timepoint 1 and timepoint 2 were co-registered with the 3D anatomical surface generated from the mean anatomical value for each subject by FreeSurfer (Version 6.0) for each participant and mapped onto a common structural template for group analysis using isomorphic 2 mm voxels. Functional data were preprocessed using the standard FS-FAST BOLD processing pipeline interoperating with FSL (Version 5.0) to apply motion-correction, slice-time correction and spatial smoothing using a 2 mm Gaussian kernel. For the functional connectivity estimation, an additional voxel-wise detrending step removed linear trends and regressed out white matter and CSF signal from the data.

### GLM Analysis and Region of Interest Construction

Subject-level GLM analyses were performed for each participant in the Reading Set, combining the functional data from both longitudinal timepoints. The event-related GLM analyses used the SPM canonical hemodynamic response function to model blood oxygen dependent (BOLD) responses for the lexical, fixation cross baseline, and symbol matching trials. Subject-level contrasts between lexical trials and fixation cross baseline were carried out separately for word and pseudoword runs. Group-level random effects analyses concatenated the first level analyses for poor- and highly-skilled readers separately. These single-sample group-level contrast maps allowed unbiased selection of regions involved in either word or pseudoword processing for either poor or highly-skilled readers.

Large cortical patches are unlikely to be homogenously organized, and so custom Python scripts, written by the author, computed the union of the group-level cluster map cortical surface annotation files that was then intersected with the FreeSurfer surface annotation of the Destrieux et al. (2010) atlas. This procedure subdivided functional clusters along anatomical boundaries into multiple regions of interest (ROI). Large ROIs that remained after this subdivision were manually subdivided into smaller regions of visually similar size to other ROIs. The union of clusters from the four significant contrast maps were thus subdivided into 115 ROIs of comparable size (*M* = 145 mm^2^) to the Lausanne parcellation ROIs used in previous studies of functional connectivity in surface space (Hagmann et al., 2008, 2010; Honey et al., 2009; McNorgan and Joanisse, 2014; McNorgan et al., 2020a).

### Classifier Training

#### Pattern Generation

Classifier input patterns were generated from task-related functional connectivity among ROIs within residualized BOLD time series data for each functional run (4 pseudoword, 4 word). An initial regression removed linear trends and white matter and CSF signal, and the mean BOLD time series was computed across the voxels in each of the ROIs. The weights in a machine learning classifier are free parameters that are iteratively adjusted to fit the training data. An overabundance of free parameters can allow the classifiers to *overfit* the training data, “memorizing” the patterns rather than learning rules that generalize to novel cases. Overfitting is measured by the degree of discrepancy between training set fit and validation set fit: A model has overfit the data if it demonstrates high training set accuracy but poor validation set accuracy. Machine learning approaches commonly mitigate overfitting by increasing the number of *distinct* patterns in the training set through data augmentation (Lemley et al., 2017). This increases the ratio of unique patterns to the dimensionality of the feature encodings, introducing additional noise in the process, but providing additional contexts in which to identify reliably predictive features (see Koistinen and Holmström, 1991, for a discussion of the utility of noise in classifier training).

Data were augmented by splitting the 6-min time series in half, and computing weighted connectivity between time series vectors separately for the first and second half of each run using two methods: Pearson correlation, which measures a linear dependency between time series vectors, and cross-mutual information (XMI), which is sensitive to the general dependency between two variables, which may or may not be linear (Li, 1990), may be more sensitive to synchronization in noisy systems (Paluš, 1997), and has been shown to be a useful connectivity-based predictor in classifier-based studies of learning difficulty (McNorgan et al., 2020b). The main diagonal of the 115 × 115 symmetric matrix was eliminated, as was the redundant lower triangle of the symmetric matrix. The remaining 6,555 values were normalized, and rescaled to fall between 0 and 1. It must be noted that the correlation-based and XMI-based connectivity patterns were only moderately correlated with each other (*r* = 0.42). This is important because it ensures that an accurate classification of a validation-set pattern, e.g., computed using XMI, is not attributable to training exposure to a highly-similar pattern computed over the same time series using the Pearson correlation. Rather, the classifiers were forced to identify both linear and nonlinear coactivation dynamics that are predictive of reading skill and lexicality.

The distributions of the positively-skewed functional connectivity values were made normal by application of a square-root transformation. Each connectivity pattern vector was tagged with a value indicating lexicality (0 = pseudoword; 1 = word), and a value indicating group (0 = poor reader; 1 = highly-skilled reader). The source and destination nodes of each functional connection was also noted, and the indices of the 253 functional connections between subregions of a single cluster within the functional mask were recorded for subsequent filtering, as these connections might reflect trivial correlations.

#### Classifier Model Architecture

Classifier models were implemented in *TensorFlow* (Version 2.2, https://www.tensorflow.org), using hyperparameters and a model architecture informed by a previous study performing orthogonal ADHD diagnosis and behavioral profile classification in an unrelated dataset (McNorgan et al., 2020a). Input values fed forward through a Gaussian noise (*SD* = 0.05) and a dropout layer (rate = 0.2), which further distorted the training patterns as a standard data augmentation method (Srivastava et al., 2014). The standard deviation of the Gaussian noise was selected to roughly match the standard deviation of input values (*SD* = 0.07). The perturbed input patterns fed forward through three densely connected rectified linear unit hidden layers, with the size of each layer determined by a logarithmic function (base 2) of the number of input features: The size of the first hidden layer was determined using formula (1), where *i* is the number of input features:

(1) $$\begin{array}{l} max\left(16,2\times ceil\left({\text{log}}_{2}\;i\right)\right) \end{array}$$

The second and third hidden layers were always half the size of the first hidden layer. Batch normalization was applied at each hidden layer (Ioffe and Szegedy, 2015). The first hidden layer additionally used an ℓ1 (LASSO) regularizer of 0.0005 to promote sparsity among the weights and act as a pruning mechanism (Allen, 2013) by eliminating redundant or non-predictive (i.e., noisy) features. These activations fed forward to two single-unit logistic classifier layers using the sigmoid activation function to simultaneously classify patterns with respect to condition lexicality and group. Models of this architecture are also known as multilayer perceptrons (MLP), and compute a Bayesian posterior probability of category membership, given a set of input features (Ruck et al., 1990). An important feature of multilayer networks is that the imposition of small hidden layers between the input and classifier layers requires the network to compute a low-dimensional transformation of the high-dimensional input patterns. This denoises and computes a nonlinear stochastic independent components analysis (ICA) on the input patterns (Hyvärinen and Bingham, 2003).

**Figure 1:**
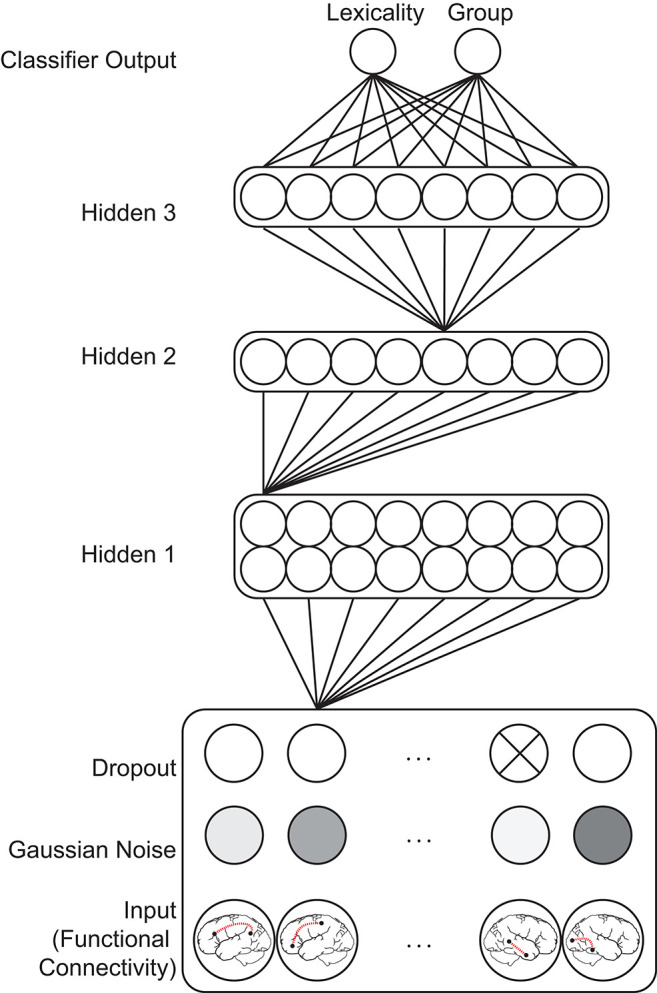
Feedforward classifier model architecture. Gaussian noise and probabilistic dropout perturbs functional connectivity input patterns, which feed forward through a series of densely connected hidden layers before reaching two classifier output units. Most model weights are omitted for clarity. The influence of a particular functional connection on a classification decision is indexed by summing the weights of all paths between the corresponding input unit and a classifier unit. Multiplication of the series of the matrices encoding the weights between each layer computes these values in parallel for all input features.

In the context of this network, classification decisions were thus made from configurations of functional connections encoded in the final hidden layer, rather than on individual functional connections encoded in the input layer as would be the case in a standard SVM classifier. This is important to bear in mind because the ratio of training examples to the dimensionality of the hidden layer ICA transformation was over 30:1. A comparison between performance of SVM and MLP single-class classification of high-dimensional connectivity vectors in McNorgan et al. (2020a) found that the SVM classifier had a propensity to overfit the training patterns, demonstrating poor validation set accuracy (58% for group classification). In contrast, the MLP classifier showed high validation set accuracy (91%) for group classification of the same data, demonstrating the utility of hidden layer ICA dimensionality reduction afforded by MLP models. The training set was balanced with respect to both classifications and the categories were orthogonal. The classifier model architecture is illustrated in Figure 1.

#### Training Parameters and Procedure

Classifier models were trained over 216 epochs using the standard gradient descent optimizer with a learning rate of ε = 0.01, decay = 0.05 and momentum = 0.9, and a batch size of 16. Early-stopping was used, monitoring lexical classifier error (Caruana et al., 2001). Stratified K-folds cross-validation (Diamantidis et al., 2000) pseudo-randomly partitioned the patterns into *K* = 5 balanced sets of training and withheld validation set data, such that each pattern appeared among the validation set data exactly once. One model was trained for each of the folds, and performance was evaluated on the withheld validation set, allowing performance metrics to reflect the model's ability to generalize to novel data. Note that the machine learning literature may distinguish between validation and test sets, with validation sets partitioned from the training data for initial model hyperparameter tuning, and test sets removed from the training data for measuring the model's ability to generalize to new data (Larsen et al., 1996), however this study refers to withheld cross-validation set accuracy as validation set performance, as hyperparameter tuning was not performed.

#### Iterative Feature Reduction

Classifier training often applies feature set reduction to eliminate uninformative features and improve accuracy (Chu et al., 2012). In addition to the nonlinear ICA imposed by the model's hidden layer architecture, feature reduction at the input layer was used as an analog to backwards stepwise linear regression in this study as a means of identifying the small proportion of functional connections with the greatest influence on classification decisions.

An iterative feature selection algorithm was applied over a series of model generations. Beginning with the full set of functional connectivity patterns, the 253 functional connections between nodes within clusters from the unified GLM contrast maps were eliminated from the input patterns prior to the first model generation. After training, cross-validation set performance was assessed for each fold, and the summed path weights from each remaining input feature unit to each of the classifier output units was computed. Each input feature unit contributes toward each classification decision by driving the classifier unit toward either 0.0 (if the summed path weights are negative) or 1.0 (if the summed path weights are positive). After all training folds had completed, the input feature set was reduced through *decimation*, by which the summed absolute value of input feature weights to both classifier units were ranked-ordered, and features that were in the bottom tenth percentile for *either* classification were eliminated for the subsequent generation. Training proceeded in this way until the full feature set had been reduced to 0.05 of its original size (i.e., finding the 95th percentile of functional connections predictive of *both* lexicality and group) while still demonstrating above-chance classification accuracy, requiring either 15 or 16 generations. This was repeated twenty times to produce a sample of *n* = 20 model families. Note that, because the hidden layer size was a logarithmic function of the number of input features, hidden layers were also reduced across generations. The final generation of models contained 0.01 of the number of trainable weights as the first generation models, despite containing 0.05 the number of input features. The disproportionately large reduction in trainable weights across model generations greatly reduced the representational capacity of later models.

The feature selection procedure leaks information about the most informative features between generations of a single family of models, however this is not problematic for several reasons: First, the relative diagnostic utility of an input feature is not fixed as the models become more constrained with the elimination of hidden units across generations. Second, feature reduction was intended to facilitate interpretation, rather than improve accuracy (it will be shown that removing 0.95 of the input data and 0.99 of the trainable model weights decreased classification accuracy); the survival and subsequent inclusion of predictor x_*i*_ in the *n*+1^th^ model generation is analogous to the survival of predictor *x*_i_ into the *n*+1^th^ step in a backwards stepwise multiple regression. Finally, each of the 20 model families are independent, precluding information leakage *between* model families. The analyses that follow aggregate results across all model families, permitting measures of predictive reliability for each functional connection, and more importantly, the evaluation of a composite model comprising the most informative features independently identified by each of the model families.

#### Model Evaluation

Each generation of models was evaluated with respect to validation set classification accuracy for lexical condition and group, and d′ was computed, collapsing across all cross-validation folds. The frequency with which each functional connection was retained in the final generation of models was summed across the twenty model families, and used to inform the construction of a composite model comprising the most informative features across all model families. Input features appeared with decreasing frequency among the final generation of models, with more than a third appearing in none of the models, and most of the remaining appearing in at most one model. The most informative features were defined as those 327 functional connections that appeared in at least 4 of 20 final-generation models, because they represented 0.049 of the full set of functional connections, and thus corresponded to the 95th percentile of most predictive features. Validation set lexicality and group classification performance was assessed for a composite model trained using this set of functional connections using 10-fold cross-validation to permit relative comparisons among the most reliably predictive features. This composite model served as a direct evaluation of these putatively predictive functional connections in isolation.

### Math Set Evaluation

Five sets of first-generation classifier models were trained using all Reading Set patterns as training data. Classification accuracy and d′ scores were computed for classification decisions on functional connectivity patterns from the Math Set mental multiplication task as a replication to bolster claims of classifier generalizability and test whether lexically-related functional connectivity within the reading network distinguishes poor from highly-skilled readers, even when engaged in a non-reading task. This evaluation followed the procedure described above for evaluating classifier models performance at the first generation, with two differences: First, only 3 cross-validation folds were used in each set—each network was trained using a random .66 partitioning of the Reading Data. The second critical difference was that, in place of the withheld Reading Data, classification performance was evaluated using patterns from the Math Set.

## Results

### GLM Lexicality Contrasts

**Figure 2:**
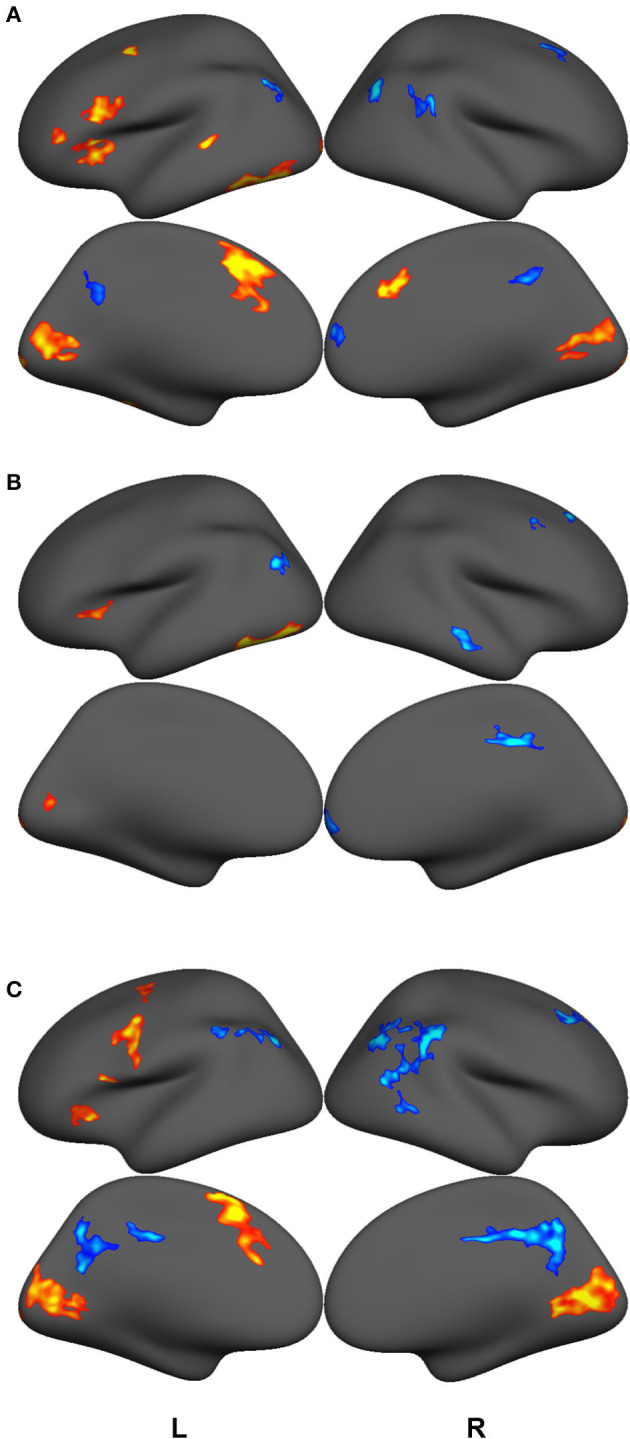
GLM contrast maps showing regions with significant activation (hot) or deactivation (cold) for lexical trials vs. fixation cross baseline for good readers making rhyming judgments on words **(A)** and pseudowords **(B)** and for poor readers making rhyming judgments on words **(C)**. All maps thresholded with a voxel-wise *p* = 0.001 and a cluster-size corrected threshold of *P* = 0.05. There were no regions showing above-threshold differences between pseudoword and fixation cross baseline among poor readers. The union of these clusters was used as a functional mask to define the Reading Network. Functional connectivity was computed over anatomically-delineated subdivisions of the Reading Network.

GLM contrast maps were thresholded using a voxel-wise significance level of *p* = 0.001 and a cluster-size threshold of *P* = 0.05 based on the FreeSurfer random permutation cluster simulation. Figure 2 and Tables 3, 4, 5 shows retained clusters showing contrasts for highly-skilled readers between words and fixation (A) and pseudowords and fixation (B), and for poor readers between words and fixation (C). Poor readers had no above-threshold regions showing significant differences between pseudowords and fixation. The union of the GLM contrast maps depicted in Figure 2 served as a functionally-defined mask that include regions with high signal-to-noise and associated with preferential responses to either words or pseudowords for either highly-skilled readers or poor readers, and functional connectivity among these regions was computed for the analyses that follow. Contrasts between lexical conditions or groups would select regions with *a priori* bias toward one condition or group and thus not performed. Similar analyses have been reported elsewhere on supersets of these data (McNorgan et al., 2014; McNorgan and Booth, 2015; Edwards et al., 2018; Smith et al., 2018), and so the GLM results are not discussed further.

**Table 3:** Coordinates of peak surface activity within clusters reaching *P* < 0.05 (cluster-level corrected) significance in the words vs. fixation contrast for highly-skilled readers.

**Contrast**	**Region**	***t***	**Size**	**X**	**Y**	**Z**	***p***
W > Fix	r. superior frontal	6.46	315	12	28	33	0.0001
	l. superior frontal	6.34	888	−11	12	43	0.0001
	l. superior temporal sulcus	5.99	155	−58	−35	3	0.0116
	l. fusiform	5.89	1383	−41	−64	−14	0.0001
	l. lateral occipital	5.83	443	−17	−99	−11	0.0001
	l. caudal middle frontal	5.68	172	−36	1	48	0.0068
	l. pars opercularis	5.61	458	−36	24	10	0.0001
	r. lateral occipital	5.50	295	21	−92	−8	0.0001
	l. pars opercularis	5.49	556	−51	17	22	0.0001
	l. pericalcarine	5.25	1118	−13	−83	4	0.0001
	r. pericalcarine	4.74	959	7	−78	12	0.0001
	l. pars triangularis	4.63	220	−46	34	4	0.0018
W < Fix	r. inferior parietal	−5.55	333	40	−71	39	0.0001
	l. inferior parietal	−5.07	274	−41	−67	39	0.0005
	r. supramarginal	−4.82	353	60	−41	22	0.0001
	r. superior frontal	−4.73	179	22	12	47	0.0049
	r. precuneus	−4.63	188	9	−43	39	0.0030
	r. superior frontal	−4.27	182	9	54	15	0.0043
	l. precuneus	−4.02	198	−13	−55	32	0.0034

*L, left; R, right; t, peak t-statistic; Size, cluster extent in mm^2^. Coordinates reflect standard MNI space. Region labels derived from the FreeSurfer Desikan-Killiany atlas surface*.

**Table 4:** Coordinates of peak surface activity within clusters reaching *P* < 0.05 (cluster-level corrected) significance in the pseudowords vs. fixation contrast for highly-skilled readers.

**Contrast**	**Region**	***t***	**Size**	**X**	**Y**	**Z**	***p***
PW > Fix	l. fusiform	8.081	936	−40	−71	−12	0.0001
	l. lateral occipital	6.268	339	−17	100	−7	0.0001
	l. lateral occipital	5.937	171	−17	−99	−8	0.0033
	l. insula	4.255	154	−28	23	5	0.0063
	l. pericalcarine	4.152	137	−12	−79	12	0.0113
PW < Fix	r. precuneus	−7.245	304	6	−37	42	0.0001
	l. inferior parietal	−5.427	272	−45	−69	25	0.0002
	r. superior frontal	−5.355	112	21	26	46	0.0138
	r. middle temporal	−5.314	287	65	−19	−15	0.0001
	r. medial orbitofrontal	−4.256	301	9	55	−4	0.0001
	r. caudal middle frontal	−4.243	113	40	10	51	0.0138

*L, left; R, right; t, peak t-statistic; Size, cluster extent in mm^2^. Coordinates reflect standard MNI space. Region labels derived from the FreeSurfer Desikan-Killiany atlas surface*.

**Table 5:** Coordinates of peak surface activity within clusters reaching *P* < 0.05 (cluster-level corrected) significance in the words vs. fixation contrast for poor readers.

**Contrast**	**Region**	***t***	**Size**	**X**	**Y**	**Z**	***p***
W > Fix	l. superior frontal	7.08	798	−10	13	52	0.0001
	l. lateral occipital	6.591	229	−17	−101	−6	0.0007
	r. pericalcarine	6.326	1863	18	−72	11	0.0001
	l. precentral	5.69	600	−52	−2	45	0.0001
	l. pericalcarine	5.312	1623	−9	−81	3	0.0001
	l. lateral orbitofrontal	5.28	308	−27	28	−3	0.0001
	l. pars opercularis	5.148	144	−53	14	14	0.0093
	l. precentral	3.867	206	−25	−14	56	0.0010
W < Fix	r. precuneus	−6.447	1064	6	−38	42	0.0001
	r. supramarginal	−6.141	1197	53	−45	28	0.0001
	l. inferior parietal	−5.713	266	−37	−74	38	0.0002
	r. rostral middle frontal	−5.323	316	27	25	38	0.0001
	l. precuneus	−5.29	616	−14	−62	23	0.0001
	r. inferior parietal	−5.289	715	41	−69	39	0.0001
	l. posterior cingulate	−5.007	176	−6	−27	39	0.0026
	l. inferior parietal	−4.665	195	−49	−55	38	0.0015
	r. middle temporal	−4.35	188	53	−55	−2	0.0013
	l. supramarginal	−4.3	180	−56	−45	40	0.0023

*L, left; R, right; t, peak t-statistic; Size, cluster extent in mm^2^. Coordinates reflect standard MNI space. Region labels derived from the FreeSurfer Desikan-Killiany atlas*.

### Classifier Performance

Reading Set validation set classification accuracy and d′ was computed separately for lexicality and group classifications among the twenty families of models. These values were computed separately for each lexicality and group, however because the 95% confidence intervals overlapped between words and pseudowords and between poor and very skilled readers in each analysis, both lexicality conditions and both group conditions were collapsed in the presentation of results that follow.

### First Generation

Among all first-generation models, which used all but the 253 short-distance within-cluster functional connections as predictors, lexical classification accuracy (*M* = 0.64, *SD* = 0.04,0.95 CI [0.63, 0.66]) was well above chance, with all chi-squared tests of the ratios of classification decisions to expected chance performance significant at *p* < 10^−21^, and mean d′ of 0.74, 0.95 CI [0.65, 0.83]. The mean phi coefficients of the contingency table for each model family was 0.29, *SD* = 0.09,0.95 CI [0.25, 0.32], indicating that middle- to long-distance task-dependent functional connections within the reading network bear a weak- to moderate explanatory relationship with lexical task.

Group classification accuracy (*M* = 0.94, *SD* = 0.06.95 CI [0.92,0.96]) was high for all first-generation model families, with all chi-squared tests of the ratios of classification decisions to expected chance performance significant at *p* < 10^−256^, and mean d′ of 3.58, 0.95 CI [3.28, 3.88]. The mean phi coefficient of the contingency table for each model family was 0.88, *SD* = 0.11, 0.95 CI = [0.84, 0.93], indicating that middle- to long-distance task-dependent functional connections within the reading network bear a very strong explanatory relationship with reading skill.

### Final Generation

Among all final-generation models, lexical classification accuracy (*M* = 0.53, *SD* = 0.01.95 CI [0.52, 0.53]) was above chance for most models, with 18 of 20 chi-squared tests of classification accuracy significant at *p* < 0.05, and a mean d′ of 0.13,0.95 CI [0.11, 0.15]. The mean phi coefficients of the contingency table for each model family was 0.05, *SD* = 0.02,0.95 CI = [0.04, 0.06], indicating that the top 5% most predictive middle- to long-distance task-dependent functional connections within the reading network bear a negligible explanatory relationship with lexical task.

Group classification accuracy (*M* = 0.71, *SD* = 0.03.95 CI [0.70, 0.72]) remained well above chance for all final-generation model families, with all chi-squared tests of the ratios of classification decisions to expected chance performance significant at *p* < 10^−110^, and mean d′ of 1.22,0.95 CI [1.15, 1.29]. The mean phi coefficient of the contingency table for each model family was 0.42, SD = 0.06,0.95 CI = [0.40, 0.44], indicating that the 0.05 most informative middle- to long-distance task-dependent functional connections among the reading network bear a strong explanatory relationship with reading skill.

**Figure 3:**
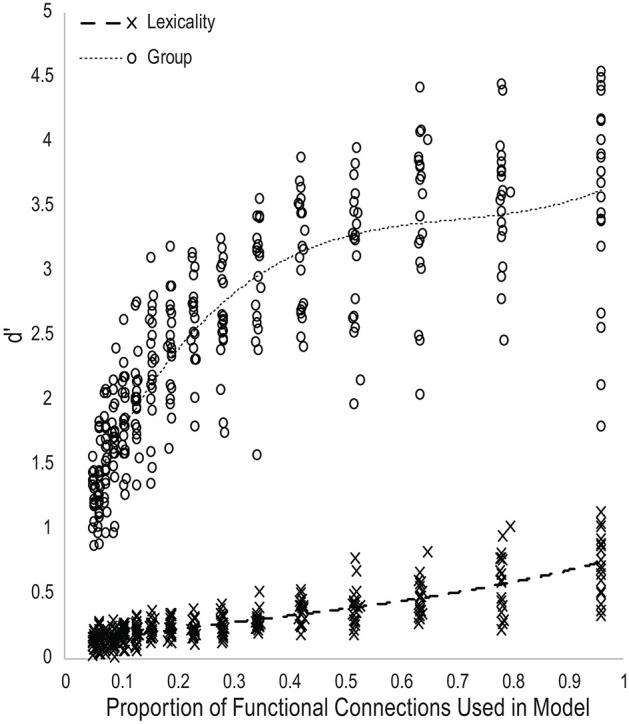
Scatterplot of d-prime measures of classifier performance for each generation of twenty model families as a function of the proportion of the total number of functional connections used to predict reading skill group or lexicality.

Figure 3 plots the d′ scores for lexical and group classification performance for all models as a function of the proportion of the total number of functional connections used as input features. Trendlines were fit with a third-order polynomial. The performance trajectory shows a nearly linear relationship between lexicality classification performance and the number of functional connections used in the models, suggesting that most functional connections within the task-defined network are roughly equally predictive of lexicality condition, and thus that classification accuracy is proportional to the number of connections in the model. The performance trajectory for group classification shows a nearly logarithmic relationship, with a large increase in classification performance between models using 0.05 and 0.15 of all functional connections, and declining gains in accuracy as models become larger. This indicates that a small number of functional connections within the task-defined network are disproportionately predictive of reading skill. Finally, d′ measures of lexicality and group classification performance were positively correlated across all models, r_(315)_ = 0.71, *p* < 10^−48^. As would be expected by the equal weighting given to the error signal for the two classifier units, this indicates that optimizing accuracy in one classification decision was not at the expense of the other.

### Evaluation of Predictive Functional Connections Using a Composite Model

Of the 6,555 functional connections, 2,770 never appeared in a final model, with most of the remaining features appearing in at most one of the 20 final-generation models. Validation set lexicality and group classification performance was assessed for a composite model trained using the set of 327 functional connections appearing in at least 4 final generation models using 10-fold cross-validation to permit relative comparisons among the most reliably predictive features, which were otherwise found in independent model families. Collapsed across all 10 model folds, the d' measure was 0.03 for lexical classification accuracy, but 1.02 for group classification accuracy, which was significantly better than chance, $X_{\left(1\right)}^{2}$ = 58.58, *p* < 10^−13^, and indicates that the 95th percentile of most predictive functional connections within the task-defined network are significant predictors of reading skill.

**Figure 4:**
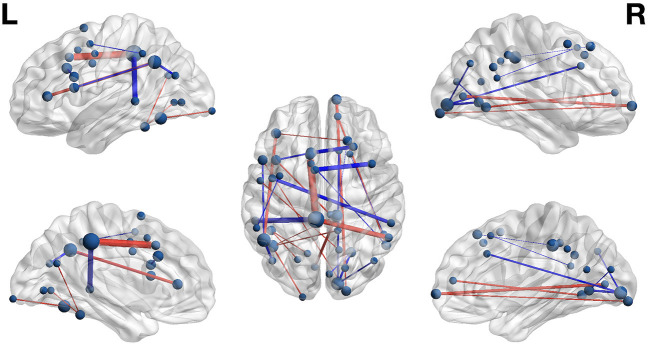
Functional networks including only those highly-relevant functional connections where high connectivity was predictive of poor (blue) or highly-skilled (red) reading in the set of composite models. Edge thickness indexes connectivity strength, and ROI node diameter indexes summed connectivity with other regions.

**Figure 5:**
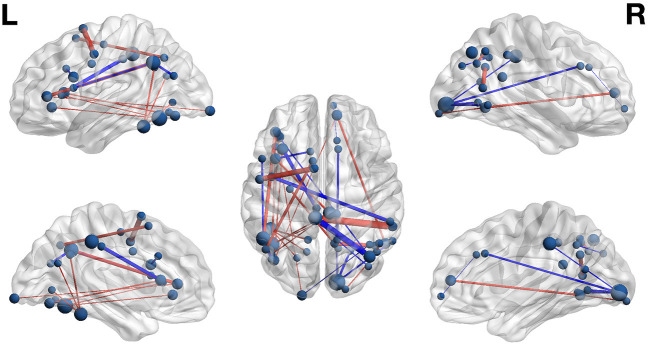
Functional networks including only those highly-relevant functional connections where high connectivity was predictive of pseudoword (blue) and word (red) lexicality condition in the set of composite models. Edge thickness indexes connectivity strength, and ROI node diameter indexes summed connectivity with other regions.

The weight structures of the composite models generated by each fold were used to construct functional networks for further analysis and visualization: The magnitude of a path weight indexes the relevance of that feature on the classification decision, and the valence of the weight indicates the classification associated with high functional connectivity (negative: pseudoword, poor reader; positive: word, highly-skilled reader). To limit the scope of analysis, the weight magnitudes were normalized, and those functional connections with |Z| > 1.0 were selected as *highly-relevant*. To enable visualization of networks comprising only highly relevant connections, adjacency matrices were constructed for functional networks predicting group and lexicality classification by adding to an empty adjacency matrix the corresponding mean functional connectivity score for all highly-relevant functional connections identified in the previous step. For example, high connectivity between left inferior frontal sulcus and right cingulate ROIs was a highly-relevant predictor of the highly-skilled reading group. The mean connectivity between these regions for all run splits for all highly-skilled readers was thus added to the Group adjacency matrix. These adjacency matrices were used to generate Figures 4, 5.

### Group Classification

Functional connectivity networks including only those functional connections with strong classification weights for reading skill are rendered in Figure 4 and Table 6. From this figure are highlighted regions that participate in multiple predictive functional connections, and therefore may be important network hubs. Three adjacent nodes belonging to a functional cluster spanning the right occipital pole and right calcarine sulcus were terminal points of multiple functional connections within the right hemisphere that are predictive of poor reading skill. Similarly, two adjacent nodes belonging to a functional cluster within the left occipitotemporal cortex were terminal points of multiple functional connections both within and between hemispheres that are predictive of high reading skill. Finally, two regions of interest, one within the left precuneus and the other within the anterior intermediate parietal sulcus of Jensen, were terminal points of the functional connections that most reliably predict *both* poor and highly-skilled reading, suggesting that these regions may be critical hubs supporting reading.

**Table 6:** Highly relevant functional connections predictive of reading skill in composite model.

**Group**	**Source region**	**X**	**Y**	**Z**	**Destination region**	**X**	**Y**	**Z**	***r***	***n***
Poor Skill	l Cingulate Gyrus	−10	20	30	l Inferior Frontal Sulcus	−39	14	23	0.52	9
	l Cingulate Gyrus	−10	20	30	r Superior Frontal Sulcus	25	25	41	0.64	8
	l Jensen Sulcus	−49	−52	35	l Pars Opercularis	−50	14	14	0.52	10
	l Jensen Sulcus	−49	−52	35	l Superior Temporal Sulcus	−42	−69	24	0.55	4
	l Precentral Sulcus	−42	−1	34	r Supramarginal Gyrus	56	−38	41	0.62	9
	l Precuneus	−6	−35	42	l Superior Temporal Sulcus	−56	−36	3	0.72	8
	l Superior Frontal Gyrus	−10	11	45	r Inferior Temporal Sulcus	52	−56	−2	0.47	10
	l Supplementary Motor Area	−7	6	63	r Middle Frontal Gyrus	40	11	50	0.69	5
	l Supramarginal Gyrus	−55	−42	42	l Middle Frontal Gyrus	−36	1	50	0.47	7
	r Cuneus	5	−80	19	r Calcarine Sulcus	23	−62	2	0.48	5
	r Occipital Pole	12	−88	0	r Angular Gyrus	42	−69	34	0.50	7
	r Occipital Pole	12	−88	0	r Anterior Cingulate Gyrus	13	22	32	0.52	11
	r Occipital Pole	12	−88	0	r Calcarine Sulcus	23	−62	2	0.54	4
	r Superior Frontal Gyrus	20	30	48	r Marginal Sulcus	11	−32	39	0.43	6
	r Superior Frontal Gyrus	22	19	47	r Superior Temporal Sulcus	50	−47	22	0.44	4
High Skill	l Inferior Frontal Sulcus	−39	14	23	r Cingulate Gyrus	6	−31	40	0.50	6
	l Inferior Frontal Sulcus	−39	36	7	r Superior Frontal Gyrus	20	30	48	0.47	5
	l Jensen Sulcus	−49	−52	35	l Inferior Frontal Sulcus	−39	36	7	0.59	7
	l Lateral Occipitotemporal Gyrus	−42	−57	−11	l Occipital Pole	−17	−99	−5	0.49	6
	l Lateral Occipitotemporal Gyrus	−42	−57	−11	r Subparietal Sulcus	12	−44	36	0.48	6
	l Lateral Occipitotemporal Gyrus	−43	−44	−18	l Calcarine Sulcus	−21	−68	2	0.47	4
	l Lateral Occipitotemporal Gyrus	−43	−44	−18	l Parieto–occipital Sulcus	−13	−63	24	0.47	6
	l Lateral Occipitotemporal Gyrus	−42	−57	−11	r Marginal Sulcus	11	−32	39	0.46	5
	l Medial Lingual Gyrus	−7	−74	2	r Marginal Sulcus	11	−32	39	0.57	9
	l Pars Opercularis	−50	14	14	r Occipital Pole	19	−94	−7	0.47	7
	l Precentral Gyrus	−52	−3	42	r Parieto–occipital Sulcus	13	−60	19	0.47	7
	l Precuneus	−6	−35	42	l Cingulate Gyrus	−11	18	39	0.84	6
	l Precuneus	−6	−35	42	r Angular Gyrus	54	−50	31	0.71	8
	r Calcarine Sulcus	18	−75	7	r Transverse Frontopolar Gyrus	11	64	−1	0.59	8
	r Inferior Temporal Sulcus	52	−56	−2	r Anterior Cingulate Gyrus	12	50	10	0.52	10
	r Occipital Pole	19	−94	−7	r Transverse Frontopolar Gyrus	11	64	−1	0.49	7

*L, left; R, right; n, number of models (/20) containing connection; Coordinates reflect standard MNI space. Region labels derived from the FreeSurfer, Destrieux et al. (2010) atlas*.

### Lexicality Classification

The functional connectivity network including only those functional connections with strong classification weights for lexical condition are rendered in Figure 5 and Table 7. It is notable that that the set of highly-relevant functional connections was dominated by multiply-connected hubs within the left hemisphere, many along the left occipitotemporal sulcus, consistent with the privileged role of this region in orthographic processing. However, because lexicality classification among composite models was at chance accuracy, this network should be interpreted with caution: Though the identified connections are the *most* individually predictive of lexicality, they are only a subset of the functional connections that accurately predict lexicality. This follows from the observation that iterative pruning produced a linear decrease in classification accuracy. Thus, the core functional networks supporting word and pseudoword processing appear to be very similar, and become reliably differentiable only when examining connectivity among the reading network as a whole.

**Table 7:** Highly relevant functional connections predictive of lexicality in composite model.

**Lexicality**	**Source region**	**X**	**Y**	**Z**	**Destination region**	**X**	**Y**	**Z**	***r***	***n***
Pseudoword	l Anterior Lateral Fissure	−36	32	−2	r Angular Gyrus	39	−67	43	0.46	7
	l Cingulate Gyrus	−10	20	30	l Inferior Frontal Sulcus	−39	14	23	0.49	9
	l Jensen Sulcus	−49	−52	35	l Superior Temporal Sulcus	−42	−69	24	0.52	4
	l Occipital Pole	−17	−99	−5	r Intraparietal Sulcus	39	−57	41	0.44	6
	l Pars Opercularis	−50	14	14	l Jensen Sulcus	−49	−52	35	0.49	10
	l Posterior Cingulate Gyrus	−5	−27	38	l Insula	−33	22	10	0.59	7
	l Precentral Sulcus	−42	−1	34	r Supramarginal Gyrus	56	−38	41	0.59	9
	l Precuneus	−6	−35	42	r Angular Gyrus	39	−67	43	0.72	7
	r Anterior Cingulate Gyrus	12	29	31	r Superior Frontal Gyrus	9	58	−3	0.41	5
	r Occipital Pole	12	−88	0	r Calcarine Sulcus	23	−62	2	0.51	4
	r Occipital Pole	12	−88	0	r Anterior Cingulate Gyrus	13	22	32	0.5	11
	r Occipital Pole	12	−88	0	r Middle Temporal Gyrus	58	−54	0	0.48	6
	r Occipital Pole	12	−88	0	r Cingulate Marginalis	11	−32	39	0.45	4
	r Subparietal Sulcus	12	−53	39	r Middle Occipital Gyrus	38	−75	32	0.45	5
Word	l Inferior Frontal Sulcus	−39	36	7	l Jensen Sulcus	−49	−52	35	0.57	7
	l Inferior Frontal Sulcus	−39	14	23	r Posterior Cingulate Gyrus	6	−31	40	0.49	6
	l Inferior Frontal Sulcus	−39	36	7	l Occipital Pole	−17	−99	−5	0.43	4
	l Inferior Temporal Sulcus	−42	−65	−6	r Posterior Cingulate Gyrus	6	−31	40	0.45	4
	l Occipital Sulcus	−40	−70	−9	l Insula	−33	22	10	0.46	5
	l Occipital Sulcus	−40	−70	−9	r Anterior Cingulate Gyrus	12	50	10	0.43	5
	l Occipitotemporal Sulcus	−43	−44	−18	l Subparietal Sulcus	−12	−53	31	0.48	4
	l Occipitotemporal Sulcus	−42	−57	−11	r Posterior Cingulate Gyrus	6	−31	40	0.46	6
	l Occipitotemporal Sulcus	−43	−44	−18	l Calcarine Sulcus	−21	−68	2	0.45	4
	l Occipitotemporal Sulcus	−43	−44	−18	l Parieto–occipito Sulcus	−13	−63	24	0.45	6
	l Occipitotemporal Sulcus	−42	−57	−11	l Lateral Fissure	−36	32	−2	0.44	5
	l Occipitotemporal Sulcus	−42	−57	−11	l Lateral Fissure	−39	24	8	0.44	5
	l Occipitotemporal Sulcus	−42	−57	−11	r Cingulate Marginalis	11	−32	39	0.44	5
	l Precentral Sulcus	−27	−11	50	r Parieto–Occipital Sulcus	13	−60	19	0.5	7
	l Precuneus	−6	−35	42	r Supramarginal Gyrus	58	−42	23	0.78	7
	l Superior Frontal Gyrus	−8	12	55	l Angular Gyrus	−47	−61	39	0.54	5
	l Supplementary Motor Area	−7	6	63	l Precentral Gyrus	−52	−3	42	0.68	8
	r Angular Gyrus	47	−56	44	r Angular Gyrus	45	−63	34	0.52	6
	r Inferior Temporal Sulcus	52	−56	−2	r Anterior Cingulate Gyrus	12	50	10	0.5	10
	r Occipital Pole	19	−94	−7	r Inferior Temporal Sulcus	52	−56	−2	0.45	6
	r Subparietal Sulcus	10	−56	31	r Superior Temporal Sulcus	44	−58	14	0.62	7

*L, left; R, right; n, number of models (/20) containing connection; Coordinates reflect standard MNI space. Region labels derived from the FreeSurfer, Destrieux et al. (2010) atlas*.

### Math Dataset

Across all five sets of models, classification accuracy was comparable to classification of the Reading Dataset (*M* = 0.96, *SD* = 0.04, 0.95 CI = [0.92, 0.98]), with all chi-squared tests of the ratios of classification decisions to expected chance performance significant at *p* < 10^−18^, and mean d′ score of 5.60 (*SD* = 1.42;0.95 CI = [4.25, 6.95]). The mean phi coefficient for the 5 model families was .91 (*SD* = 0.07;0.95 CI = [0.84, 0.98]), indicating that functional connectivity during mental multiplication within the reading network has a very strong explanatory relationship with reading skill.

There was no clear correct lexicality classification for mental multiplication connectivity patterns, and so it was expected that the lexicality classifications would be random and equiprobable. It was thus noteworthy that all models classified 100% of mental multiplication patterns as consistent with pseudoword processing.

## Discussion

This study used a classifier model in a novel analytic approach that identified functional connectivity patterns that simultaneously distinguished word from pseudoword processing with above chance accuracy and distinguished between patterns from poor and highly-skilled readers with near-ceiling accuracy, sensitivity, and specificity. Indeed, because each participant contributed multiple patterns, if the modal classification decision was used for each participant, the probability of misclassifying a participant on 5 of 8 task runs is < 10^−5^. The phi coefficient is a non-parametric relative of the Pearson correlation, measuring the strength of association between two variables. To provide context for the phi coefficients computed from the classification of reading skill in both the Reading and Math sets, they are well above the correlations reported between reading skill and predictors such as working memory (*r* = 0.29; Peng et al., 2018), or phonological processing, which has been argued to play a causal role in reading acquisition (*r* = 0.09 to 0.73 depending on measure; Wagner, 1988). The results indicate that a functional connectivity fingerprint of reading skill can be found within the network supporting word and pseudoword reading, and produced two unexpected findings not discoverable by traditional univariate parametric approaches.

### Reading Skill Shapes Functional Organization in Other Cognitive Domains

First, task-based functional connectivity fingerprints of poor- and highly-skilled reading are present in task-based functional connectivity in other cognitive domains. Functional networks are dynamic, reflecting the demands of the cognitive task (Gonzalez-Castillo and Bandettini, 2018), and so it is remarkable that the manner in which the brain organizes during mental multiplication reflects an individual's reading skill. As noted earlier, reading difficulty is often comorbid with math difficulty, and the co-occurrence of math difficulty with reading difficulty is attributed to reliance on shared components (Fletcher, 2005). The mental multiplication task presented problems symbolically, rather than as word problems. However, the classifiers were required to simultaneously classify reading skill and lexicality, and the shared weight structure for these classifications ensured that the functional connections that discriminate reading skill are prima facie relevant to lexical processing (and vice versa). This makes it difficult to explain this finding through appeals to non-lexical processes. The single-digit multiplication task was selected because it is often taught by rote memorization, so that these problems are solved by retrieving verbalized facts, rather than algorithmically. This result may thus reflect reading-skill dependent differences in the networks that are engaged whenever accessing lexicalized knowledge.

### Lexicality Sensitivity Differentiates Poor- and Highly-Skilled Readers

Second, iterative pruning on the basis of diagnosticity for both classification decisions provided novel insight into lexical processing in poor and skilled readers. There were equal numbers of poor and highly-skilled readers, and all participants performed both word and pseudoword reading, and it was thus expected that characteristic functional connections associated with both word and pseudoword reading would be found for both groups. Instead, among the most categorically-diagnostic functional connections in the composite model, there were no cases where strong functional connectivity was strongly predictive of both poor reading skill and of word reading. Conversely, there were no cases where strong functional connectivity was strongly predictive of both high-reading skill and of pseudoword reading. This suggests that poor- and highly-skilled readers might be best differentiated by how they process pseudowords and familiar words, respectively. The most transparent explanation is that there are functional networks that are used almost exclusively by children with reading difficulty when encountering unfamiliar letter strings, and other functional networks used almost exclusively by highly-skilled readers for decoding known words. This informs the interpretation of previous network studies of normal and impaired reading (Fraga González et al., 2016; Edwards et al., 2018), suggesting that reading-skill related differences in graph-theoretic metrics of functional networks reflect different network configurations, rather than the same networks used to different extents. The absence of characteristic connectivity predicting poor reading and word processing suggests that when dyslexics encounter highly-practiced words, they use the same networks as do their non-impaired counterparts.

Poor reading skill was predicted by high connectivity between right occipital pole and right anterior cingulate and right calcarine sulcus that was also predictive of pseudoword reading. This highlights a right-lateralized network preferentially recruited by poor readers when encountering unfamiliar lexical strings, and may indicate that the posterior right hemisphere compensatory mechanism proposed by Shaywitz and Shaywitz (2005) reflects ongoing processing of lexical items after initial failed lexical retrieval. High-skilled reading was predicted by strong connectivity from two hubs in the left lateral occipitotemporal gyrus that were also predictive of word reading. This is consistent with the privileged role this region plays in orthographic processing and provides a connectivity-based explanation for the under activation of this region in dyslexics (Richlan et al., 2009). The pattern indicates that activity in this region is less coordinated with other regions in the reading network for poor readers, and also suggests precocious specialization of the region is accompanied by strong coherence with upstream left visual cortex in strong readers.

This pattern is interesting in the context of both the Price and Devlin (2011) Interactive Account and the hierarchical organization model proposed by Dehaene et al. (2005). The Interactive Account assumes that connectivity between occipitotemporal and higher-level processing regions is experience-dependent, but that connectivity with earlier visual cortex is not. Dehaene's hierarchical account argues that this region represents complex conjunctions of visual features represented in earlier visual cortex. In both of these models, strong connectivity with earlier visual cortex should support a robust representation of orthographically-relevant visual information and promote reliable orthography-phonology mapping for higher-skilled reading processing known words, consistent with the pattern-separation mechanism described by McNorgan et al. (2011).

The left Jensen sulcus, which bounds the angular and supramarginal gyri at the posterior end of the superior temporal sulcus, was the terminus of functional connections predictive of both poor reading and highly-skilled reading, and of word and pseudoword reading, depending on the connected region. Shahin et al. (2009) proposed that a circuit between left angular gyrus and superior temporal sulcus was part of a phonological repair network. Moreover, Del Tufo and Myers (2014) found that dyslexics with greater reading difficulty were more likely to engage the phonological repair processes when listening to distorted speech sounds. This connection was predictive of pseudoword reading and poor reading skill, and in this light, suggests that a dyslexic reader's difficulty mapping from orthography to phonology initiates two parallel processes: the engagement of a left hemisphere circuit to clean up a phonological representation to find a most likely match, and the engagement of a posterior right hemisphere visual circuit that facilitates processing unrecognized orthographic forms. The inferior frontal sulcus has been argued to be involved in both semantic and phonological processing (Poldrack et al., 1999; Turkeltaub et al., 2003), and high connectivity between Jensen sulcus and this posterior frontal region was predictive of highly-skilled reading of familiar words. This suggests that the rapid transmission of phonological information between left temporoparietal cortex and posterior frontal cortex for semantic and higher-order phonological analysis is a characteristic feature of highly-skilled reading.

### Other Theoretical Implications

#### The Primacy of Left Occipitotemporal Cortex in Visual Word Identification

Though brain-based theories of reading may disagree on the mechanisms underlying its privileged role in lexical processing, it is widely accepted that the left occipitotemporal cortex plays a critical role in decoding written words. Though connectivity within this region was excluded from the classifiers, multiple functional connections for which strong connectivity was predictive of word reading terminated in the left occipitotemporal sulcus, suggesting that strong functional connectivity with this region is associated with lexical decoding of familiar words. It follows that functional connectivity to and from the left occipitotemporal sulcus must be weaker when decoding unfamiliar letter strings, perhaps as a consequence of incoherent inter-regional activation patterns when pattern matching fails. The predictive reciprocal backwards connectivity to left calcarine sulcus and forwards connectivity to the inferior frontal gyrus maps well on to the circuit proposed in the Interactive Account (Price and Devlin, 2011) and those proposed to be responsible for the specialization of the visual word form area (Bouhali et al., 2014), and suggests that lexical decoding of familiar words relies on multiple intact pathways involving this region (Richardson et al., 2011).

#### Reading on a Continuum

By focusing on the extremes of the reading skill continuum, this study avoided ambiguous cases falling close to the category boundary. The multilayer classifier architecture used in this study supports multiclass categorization, allowing for classification of poor, typical and highly-skilled readers. However, though the poor readers had either diagnosed reading difficulty or received reading intervention, the highly-skilled readers did not come from an identified special population, and the extent to which this group deviates from typical readers is unknown. If reading skill is on a continuum, the inclusion of typical readers, as a midpoint between the extremes, would provide additional context for interpreting predictive connections as characteristic of a particular level of reading skill, as opposed to merely absent in one group or another. However, model performance should decrease with increasing similarity between adjacent groups.

It was hypothesized that the classifier would distinguish between opposite ends of a continuum of reading skill, however it was surprising that between-group classification of reading skill was far more accurate than was within-subject classification of lexicality. Classification in these models depends on overall pattern similarity, rather than on a mean difference threshold as in conventional parametric null hypothesis testing, and accuracy decreases when there is no clearly-matching category prototype. The original studies used an event-related design, and functional connectivity was thus computed over time series including both lexical and non-lexical trials. Non-lexical trials were common to both lexicality conditions and comprised roughly half the time series. Thus, functional connectivity was likely more similar between word and pseudoword runs than would be the case if computed over homogeneous blocks of lexical trials. This similarity should contribute toward confusability between word and pseudoword patterns. That said, task overlap alone cannot account for poorer lexicality classification because poor and highly-skilled readers completed identical tasks, yet the classifiers distinguished groups with near-ceiling accuracy. This implies that the functional connectivity patterns characteristic of reading skill are detectable even when functional connectivity measures are substantially influenced by a non-lexical task, confirmed by the near-ceiling classification accuracy of the mental multiplication task.

The overall pattern of results therefore suggests that word and pseudoword reading entails very similar processes, leading to similar category prototypes; that word and pseudoword processing is highly variable, leading to multiple prototypical patterns for each category; or possibly both. However, the results also indicate that poor and highly-skilled readers engage in characteristic processing that leads to easily-identified functional connectivity patterns. Again, the capacity to make multiclass categorizations open up the possibility of further exploration including nonwords in addition to words and pseudowords to further disentangle the networks involved in lexicality processing.

As noted earlier, there was no obvious lexical category for the mental multiplication trials, which used neither words nor pseudowords. It was thus expected that random lexicality classifications of these patterns would produce approximately equal numbers of word and pseudoword categorizations. Instead, all classifiers categorized all mental multiplication patterns as pseudowords, indicating that the functional networks recruited during mental multiplication are consistent with those used during pseudoword processing. Pseudowords are lexical strings using legal arrangements of known orthographic symbols, but with no associated semantic content. Mental multiplication problems (e.g., “3 × 5”) are likewise composed of legal arrangements of orthographic symbols with no semantic content. One explanation for this unexpected finding is that the lexical classifiers associated pseudowords with connectivity patterns related to low-level lexical syntax matching in the absence of top-down semantic input, though this implies different syntax-matching processes under semantic contexts. Additional study using experimental tasks that include nonwords and illegal mathematical expressions is required to explore this interesting cross-domain overlap.

## Limitations and Open Questions

The reported results provide distributional statistics aggregating classifier performance over hundreds of replications using a particular set of experimental hyperparameters such as learning rate, batch size, Gaussian noise distribution, dropout probability, and network size. The random nature of several of these parameters and of the training procedure itself guarantees that the network performance was not contingent on a specific set of parameters and sequence of training events, but it is tempting to speculate that a different set of parameters might produce a different outcome. This is certainly the case, though just as it is trivially easy to select an inappropriate statistical test or experimental design, it is not interesting to observe that a poorly-selected set of hyperparameters can produce a poorly-performing set of models that lead to a different conclusion. Hyperparameter optimization techniques exist (e.g., Knudde et al., 2017), and it is possible that better performance is possible. However, because the data used for hyperparameter optimization should be removed from the training set, these techniques are more data and time intensive. As described earlier, network hyperparameters were based on those from the classifier used in McNorgan et al. (2020a), and the high classification performance did not justify the additional effort. Many hyperparameter changes will have uninteresting consequences, such as changing the amount training required to reach asymptotic performance, however interesting insights might be gained from parametric manipulations to, e.g., the number of hidden units or hidden layer regularization, that impact the model's ability to encode network motifs among the hidden units.

This study used intact groups, and though the poor readers had normal-range IQ (>100) consistent with a specific reading impairment, highly-skilled readers had significantly greater scores on all IQ subtests. The set of participants within the Math Set with non-overlapping reading skill likewise differed on all IQ subtests, raising the question of whether the classifiers were categorizing on the basis of general intelligence. Though the influence of general intelligence cannot be ruled out, parallel lexicality classification using shared parameters ensured that diagnosticity was contingent on relevance to lexicality, which is confirmed by the nonrandom lexicality classification of patterns from both datasets. Intelligence is generally viewed as a complex multidimensional construct, and though poor and highly-skilled readers may differ with respect to traits like working memory that correlate with both general intelligence and reading skill, the rhyming task on which the classifiers were trained cannot be argued to be representative of general cognitive processing. It is thus probable that the models learned the connectivity fingerprints of reading skill, rather than of general intelligence level. Multiple aspects of the classifier training—from the ICA reduction of input dimensionality to the parallel within- and between-subject classification decisions—prevented the reliance on idiosyncratic (i.e., participant-specific) features extracted from training patterns. Though cross-validation measures on the reading set established classifier generalizability, cross-validation using the mental multiplication task replicated the finding and generated novel insight into the relationship between lexical processing and other cognitive tasks. The shared processing elements between the tasks remain unknown, and future studies should explore the extent to which reading-skill related connectivity influences brain processing dynamics in other cognitive tasks or the default mode network.

Finally, as with any study using intact groups, this correlational study cannot claim a causal relationship between the characteristic connectivity patterns and reading skill. Moreover, the most diagnostic functional connections are elements of larger patterns of predictive networks. Thus, even if causality could be established using manipulations that temporarily impact interregional communication (e.g., TMS), modification of individual pathways may be necessary but not sufficient to impact reading ability. Moreover, it is unclear how predictions made from simulated impairment should be interpreted, given that disruption of an intact network would correspond to an acquired dyslexia, which has a different profile from the developmental dyslexia that is the focus of this study (Baddeley et al., 1982). Nonetheless, hubs for multiply-predictive functional connections, such as the left Jensen sulcus, are an intriguing target for experimental manipulations better suited for exploring causal relationships underlying brain-behavior correlations that may drive reading skill.

## Conclusions

A multilayer perceptron classifier concurrently learned the functional connectivity fingerprints of poor and highly-skilled reading and of word and pseudoword processing within the functionally-defined reading network. These connectivity fingerprints were identifiable among functional connectivity measured in a mental multiplication task, and bore a very strong associative relationship with reading skill and a moderately strong associative relationship with lexicality processing. These results suggest that the manner in which reading skill reciprocally shapes functional connectivity in the reading network impacts dynamic brain organization in other cognitive domains, providing a path by which the uniquely human capacity for written language may influence human cognition in general.

## Data Availability Statement

Publicly available datasets were analyzed in this study. This data can be found here: https://openneuro.org/datasets/ds001894/versions/1.3.1; https://openneuro.org/datasets/ds001486/versions/1.2.2.

## Ethics Statement

The studies involving human participants were reviewed and approved by the Institutional Review Board of Northwestern University. Written informed consent to participate in the study was obtained from the participants' legal guardian/next of kin.

## Author Contributions

CM was solely responsible for all elements of the experiment and manuscript.

## Conflict of Interest

The author declares that the research was conducted in the absence of any commercial or financial relationships that could be construed as a potential conflict of interest.

## References

[B1] Allen G. I. (2013). Automatic feature selection via weighted kernels and regularization. J. Comp. Graph. Stat. 22, 284–299. 10.1080/10618600.2012.681213

[B2] Archibald L. M. Cardy J. O. Joanisse M. F. Ansari D. (2013). Language, reading, and math learning profiles in an epidemiological sample of school age children. PLoS ONE 8:e77463. 10.1371/journal.pone.0077463 24155959 PMC3796500

[B3] Baddeley A. D. Ellis N. C. Miles T. R. Lewis V. J. (1982). Developmental and acquired dyslexia: a comparison. Cognition 11, 185–199. 10.1016/0010-0277(82)90025-7 7198959

[B4] Bailey S. Hoeft F. Aboud K. Cutting L. (2016). Anomalous gray matter patterns in specific reading comprehension deficit are independent of dyslexia. Ann. Dyslexia 66, 256–274. 10.1007/s11881-015-0114-y 27324343 PMC5061587

[B5] Blau V. Reithler J. van Atteveldt N. M. Seitz J. Gerretsen P. Goebel R.. (2010). Deviant processing of letters and speech sounds as proximate cause of reading failure: a functional magnetic resonance imaging study of dyslexic children. Brain 133(Pt 3), 868–879. 10.1093/brain/awp308 20061325

[B6] Boets B. Op de Beeck H. P. Vandermosten M. Scott S. K. Gillebert C. R. Mantini D.. (2013). Intact but less accessible phonetic representations in adults with dyslexia. Science 342, 1251–1254. 10.1126/science.1244333 24311693 PMC3932003

[B7] Bouhali F. Thiebaut de Schotten M. Pinel P. Poupon C. Mangin J.-F. Dehaene S.. (2014). Anatomical connections of the visual word form area. J. Neurosci. 34, 15402–15414. 10.1523/JNEUROSCI.4918-13.2014 25392507 PMC6608451

[B8] Caruana R. Lawrence S. Giles C. L. (2001). Overfitting in neural nets: backpropagation, conjugate gradient, and early stopping, in Paper presented at the Advances in Neural Information Processing Systems (Como). 10.1109/IJCNN.2000.857823

[B9] Chu C. Hsu A.-L. Chou K.-H. Bandettini P. Lin C. (2012). Does feature selection improve classification accuracy? Impact of sample size and feature selection on classification using anatomical magnetic resonance images. Neuroimage 60, 59–70. 10.1016/j.neuroimage.2011.11.066 22166797

[B10] Dehaene S. Cohen L. Sigman M. Vinckier F. (2005). The neural code for written words: a proposal. Trends Cogn. Sci. 9, 335–341. 10.1016/j.tics.2005.05.004 15951224

[B11] Dehaene S. Pegado F. Braga L. W. Ventura P. Filho G. N. Jobert A.. (2010). How learning to read changes the cortical networks for vision and language. Science 330, 1359–1364. 10.1126/science.1194140 21071632

[B12] Del Tufo S. N. Myers E. B. (2014). Phonemic restoration in developmental dyslexia. Front. Neurosci. 8:134. 10.3389/fnins.2014.00134 24926230 PMC4044990

[B13] Destrieux C. Fischl B. Dale A. Halgren E. (2010). Automatic parcellation of human cortical gyri and sulci using standard anatomical nomenclature. Neuroimage 53, 1–15. 10.1016/j.neuroimage.2010.06.010 20547229 PMC2937159

[B14] Diamantidis N. A. Karlis D. Giakoumakis E. A. (2000). Unsupervised stratification of cross-validation for accuracy estimation. Artif. Intell 116, 1–16. 10.1016/S0004-3702(99)00094-6

[B15] Edwards E. S. Burke K. Booth J. R. McNorgan C. (2018). Dyslexia on a continuum: a complex network approach. PLoS ONE 13:e0208923. 10.1371/journal.pone.0208923 30557304 PMC6296514

[B16] Esposito F. Bertolino A. Scarabino T. Latorre V. Blasi G. Popolizio T.. (2006). Independent component model of the default-mode brain function: Assessing the impact of active thinking. Brain Res. Bull 70, 263–269. 10.1016/j.brainresbull.2006.06.012 17027761

[B17] Fletcher J. M. (2005). Predicting math outcomes: reading predictors and comorbidity. J. Learn. Disabil. 38, 308–312. 10.1177/00222194050380040501 16122061

[B18] Fraga González G. Van der Molen M. J. W. Žari,ć G. Bonte M. Tijms J. Blomert L.. (2016). Graph analysis of EEG resting state functional networks in dyslexic readers. Clin. Neurophysiol. 127, 3165–3175. 10.1016/j.clinph.2016.06.023 27476025

[B19] Gonzalez-Castillo J. Bandettini P. A. (2018). Task-based dynamic functional connectivity: recent findings and open questions. Neuroimage 180(Pt B), 526–533. 10.1016/j.neuroimage.2017.08.006 28780401 PMC5797523

[B20] Hagmann P. Cammoun L. Gigandet X. Meuli R. Honey C. J. Wedeen V. J.. (2008). Mapping the structural core of human cerebral cortex. PLoS Biol 6:e159. 10.1371/journal.pbio.0060159 18597554 PMC2443193

[B21] Hagmann P. Sporns O. Madan N. Cammoun L. Pienaar R. Wedeen V. J.. (2010). White matter maturation reshapes structural connectivity in the late developing human brain. Proc. Natl. Acad. Sci. U.S.A. 107, 19067–19072. 10.1073/pnas.1009073107 20956328 PMC2973853

[B22] Hoeft F. McCandliss B. D. Black J. M. Gantman A. Zakerani N. Hulme C.. (2011). Neural systems predicting long-term outcome in dyslexia. Proc. Natl. Acad. Sci. U.S.A. 108, 361–366. 10.1073/pnas.1008950108 21173250 PMC3017159

[B23] Honey C. J. Sporns O. Cammoun L. Gigandet X. Thiran J. P. Meuli R.. (2009). Predicting human resting-state functional connectivity from structural connectivity. Proc. Natl. Acad. Sci. U.S.A. 106, 2035–2040. 10.1073/pnas.0811168106 19188601 PMC2634800

[B24] Horowitz-Kraus T. DiFrancesco M. Kay B. Wang Y. Holland S. K. (2015). Increased resting-state functional connectivity of visual- and cognitive-control brain networks after training in children with reading difficulties. NeuroImage 8, 619–630. 10.1016/j.nicl.2015.06.010 26199874 PMC4506990

[B25] Hyvärinen A. Bingham E. (2003). Connection between multilayer perceptrons and regression using independent component analysis. Neurocomputing 50, 211–222. 10.1016/S0925-2312(01)00705-6

[B26] Ioffe S. Szegedy C. (2015). Batch normalization: Accelerating deep network training by reducing internal covariate shift. arXiv preprint arXiv:1502.03167.

[B27] Knudde N. van der Herten J. Dhaene T. Couckuyt I. (2017). GPflowOpt: a Bayesian optimization library using tensorflow. arXiv preprint arXiv:1711.03845.

[B28] Koistinen P. Holmström L. (1991). Kernel regression and backpropagation training with noise, in Paper presented at the Advances in Neural Information Processing Systems (Singapore). 10.1109/IJCNN.1991.170429

[B29] Korenberg M. J. Hunter I. W. (1990). The identification of nonlinear biological systems: Wiener kernel approaches. Ann. Biomed. Eng. 18, 629–654. 10.1007/BF02368452 2281885

[B30] Larsen J. Hansen L. K. Svarer C. Ohlsson M. (1996). Design and regularization of neural networks: the optimal use of a validation set, in Paper presented at the Neural Networks for Signal Processing VI. Proceedings of the 1996 IEEE Signal Processing Society Workshop (San Francisco, CA).

[B31] Lemley J. Bazrafkan S. Corcoran P. (2017). Smart augmentation learning an optimal data augmentation strategy. IEEE Access 5, 5858–5869. 10.1109/ACCESS.2017.2696121

[B32] Li W. (1990). Mutual information functions versus correlation functions. J. Stat. Phys 60, 823–837. 10.1007/BF01025996

[B33] Lytle M. N. McNorgan C. Booth J. R. (2019). A longitudinal neuroimaging dataset on multisensory lexical processing in school-aged children. Sci. Data 6:329. 10.1038/s41597-019-0338-5 31862878 PMC6925263

[B34] McNorgan C. Alvarez A. Bhullar A. Gayda J. Booth J. R. (2011). Prediction of reading skill several years later depends on age and brain region: implications for developmental models of reading. J. Neurosci. 31, 9641–9648. 10.1523/JNEUROSCI.0334-11.2011 21715629 PMC3147303

[B35] McNorgan C. Awati N. Desroches A. S. Booth J. R. (2014). Multimodal lexical processing in auditory cortex is literacy skill dependent. Cereb. Cortex 24, 2464–2475. 10.1093/cercor/bht100 23588185 PMC4128706

[B36] McNorgan C. Booth J. R. (2015). Skill dependent audiovisual integration in the fusiform induces repetition suppression. Brain Lang. 141, 110–123. 10.1016/j.bandl.2014.12.002 25585276 PMC4303511

[B37] McNorgan C. Joanisse M. F. (2014). A connectionist approach to mapping the human connectome permits simulations of neural activity within an artificial brain. Brain Connect 4, 40–52. 10.1089/brain.2013.0174 24117388

[B38] McNorgan C. Judson C. Handzlik D. Holden J. G. (2020a). Linking ADHD and behavioral assessment through identification of shared diagnistic task-based functional connections. Front. Physiol. 11:583005. 10.3389/fphys.2020.583005 33391011 PMC7773605

[B39] McNorgan C. Randazzo-Wagner M. Booth J. R. (2013). Cross-modal integration in the brain is related to phonological awareness only in typical readers, not in those with reading difficulty. Front. Hum. Neurosci. 7:388. 10.3389/fnhum.2013.00388 23888137 PMC3719029

[B40] McNorgan C. Smith G. J. Edwards E. S. (2020b). Integrating functional connectivity and MVPA through a multiple constraint network analysis. Neuroimage 208:116412. 10.1016/j.neuroimage.2019.116412 31790752

[B41] Mills J. R. Jackson N. E. (1990). Predictive significance of early giftedness: the case of precocious reading. J. Educ. Psychol. 82, 410–419. 10.1037/0022-0663.82.3.410

[B42] Mokhtari F. Hossein-Zadeh G.-A. (2013). Decoding brain states using backward edge elimination and graph kernels in fMRI connectivity networks. J. Neurosci. Methods 212, 259–268. 10.1016/j.jneumeth.2012.10.012 23142223

[B43] Morken F. Helland T. Hugdahl K. Specht K. (2017). Reading in dyslexia across literacy development: a longitudinal study of effective connectivity. Neuroimage 144, 92–100. 10.1016/j.neuroimage.2016.09.060 27688204

[B44] Norman K. A. Polyn S. M. Detre G. J. Haxby J. V. (2006). Beyond mind-reading: multi-voxel pattern analysis of FMRI data. Trends Cogn. Sci. 10, 424–430. 10.1016/j.tics.2006.07.005 16899397

[B45] Norton E. S. Beach S. D. Gabrieli J. D. E. (2015). Neurobiology of dyslexia. Curr. Opin. Neurobiol. 30, 73–78. 10.1016/j.conb.2014.09.007 25290881 PMC4293303

[B46] Paluš M. (1997). Detecting phase synchronization in noisy systems. Phys. Lett. A 235, 341–351. 10.1016/S0375-9601(97)00635-X

[B47] Peng P. Barnes M. Wang C. Wang W. Li S. Swanson H. L.. (2018). A meta-analysis on the relation between reading and working memory. Psychol. Bull 144:48. 10.1037/bul0000124 29083201

[B48] Perfetti C. A. Liu Y. Fiez J. Nelson J. Bolger D. J. Tan L.-H. (2007). Reading in two writing systems: accommodation and assimilation of the brain's reading network. Bilingualism 10, 131–146. 10.1017/S1366728907002891

[B49] Perfetti C. A. Tan L.-H. (2013). Write to read: the brain's universal reading and writing network. Trends Cogn. Sci. 17, 56–57. 10.1016/j.tics.2012.12.008 23357712

[B50] Poggio T. Mhaskar H. Rosasco L. Miranda B. Liao Q. (2017). Why and when can deep-but not shallow-networks avoid the curse of dimensionality: a review. Int. J. Auto. Comp. 14, 503–519. 10.1007/s11633-017-1054-2

[B51] Poldrack R. A. Wagner A. D. Prull M. W. Desmond J. E. Glover G. H. Gabrieli J. D. E. (1999). Functional specialization for semantic and phonological processing in the left inferior prefrontal cortex. Neuroimage 10, 15–35. 10.1006/nimg.1999.0441 10385578

[B52] Price C. J. Devlin J. T. (2011). The interactive account of ventral occipitotemporal contributions to reading. Trends Cogn. Sci. 15, 246–253. 10.1016/j.tics.2011.04.001 21549634 PMC3223525

[B53] Pugh K. R. Mencl W. E. Shaywitz B. A. Shaywitz S. E. Fulbright R. K. Constable R. T.. (2000). The angular gyrus in developmental dyslexia: task-specific differences in functional connectivity within posterior cortex. Psychol. Sci. 11, 51–56. 10.1111/1467-9280.00214 11228843

[B54] Randazzo M. Greenspon E. B. Booth J. R. McNorgan C. (2019). Children with reading difficulty rely on unimodal neural processing for phonemic awareness. Front. Hum. Neurosci. 13:390. 10.3389/fnhum.2019.00390 31798430 PMC6868065

[B55] Richardson F. M. Seghier M. L. Leff A. P. Thomas M. S. C. Price C. J. (2011). Multiple routes from occipital to temporal cortices during reading. J. Neurosci. 31, 8239–8247. 10.1523/JNEUROSCI.6519-10.2011 21632945 PMC3785141

[B56] Richlan F. (2019). The functional neuroanatomy of letter-speech sound integration and its relation to brain abnormalities in developmental dyslexia. Front. Hum. Neurosci. 13:21. 10.3389/fnhum.2019.00021 30774591 PMC6367238

[B57] Richlan F. Kronbichler M. Wimmer H. (2009). Functional abnormalities in the dyslexic brain: a quantitative meta-analysis of neuroimaging studies. Hum. Brain Mapp 30, 3299–3308. 10.1002/hbm.20752 19288465 PMC2989182

[B58] Ruck D. W. Rogers S. K. Kabrisky M. Oxley M. E. Suter B. W. (1990). The multilayer perceptron as an approximation to a Bayes optimal discriminant function. IEEE Trans. Neural Netw. 1, 296–298. 10.1109/72.80266 18282850

[B59] Rumelhart D. E. Hinton G. E. Williams R. J. (1985). Learning Internal Representations by Error Propagation. San Diego, CA: University of California San Diego. 10.21236/ADA164453

[B60] Shahin A. J. Bishop C. W. Miller L. M. (2009). Neural mechanisms for illusory filling-in of degraded speech. Neuroimage 44, 1133–1143. 10.1016/j.neuroimage.2008.09.045 18977448 PMC2653101

[B61] Shaywitz S. E. Shaywitz B. A. (2005). Dyslexia (specific reading disability). Biol. Psychiatry 57, 1301–1309. 10.1016/j.biopsych.2005.01.043 15950002

[B62] Smith G. J. Booth J. R. McNorgan C. (2018). Longitudinal task-related functional connectivity changes predict reading development. Front. Psychol. 9, 1–13. 10.3389/fpsyg.2018.01754 30283393 PMC6156257

[B63] Srivastava N. Hinton G. Krizhevsky A. Sutskever I. Salakhutdinov R. (2014). Dropout: a simple way to prevent neural networks from overfitting. J. Mach. Learn. Res. 15, 1929–1958.

[B64] Suárez-Pellicioni M. Lytle M. Younger J. W. Booth J. R. (2019). A longitudinal neuroimaging dataset on arithmetic processing in school children. Sci. Data 6:190040. 10.1038/sdata.2019.40 30835258 PMC6400102

[B65] Temple E. Poldrack R. A. Salidis J. Deutsch G. K. Tallal P. Merzenich M. M.. (2001). Disrupted neural responses to phonological and orthographic processing in dyslexic children: an FMRI study. Neuroreport 12, 299–307. 10.1097/00001756-200102120-00024 11209939

[B66] Turkeltaub P. E. Gareau L. Flowers D. L. Zeffiro T. A. Eden G. F. (2003). Development of neural mechanisms for reading. Nat. Neurosci. 6, 767–773. 10.1038/nn1065 12754516

[B67] van der Mark S. Bucher K. Maurer U. Schulz E. Brem S. Buckelmüller J.. (2009). Children with dyslexia lack multiple specializations along the visual word-form (vwf) system. Neuroimage 47, 1940–1949. 10.1016/j.neuroimage.2009.05.021 19446640

[B68] van der Mark S. Klaver P. Bucher K. Maurer U. Schulz E. Brem S.. (2011). The left occipitotemporal system in reading: disruption of focal fmri connectivity to left inferior frontal and inferior parietal language areas in children with dyslexia. Neuroimage 54, 2426–2436. 10.1016/j.neuroimage.2010.10.002 20934519

[B69] Wagner R. K. (1988). Causal relations between the development of phonological processing abilities and the acquisition of reading skills: a meta-analysis. Merrill-Palmer Q. 1982, 261–279.

[B70] Wolf R. C. Sambataro F. Lohr C. Steinbrink C. Martin C. Vasic N. (2010). Functional brain network abnormalities during verbal working memory performance in adolescents and young adults with dyslexia. Neuropsychologia 48, 309–318. 10.1016/j.neuropsychologia.2009.09.020 19782695

